# Effect of TNF-*α*-Induced Sclerostin on Osteocytes during Orthodontic Tooth Movement

**DOI:** 10.1155/2019/9716758

**Published:** 2019-06-24

**Authors:** Fumitoshi Ohori, Hideki Kitaura, Aseel Marahleh, Akiko Kishikawa, Saika Ogawa, Jiawei Qi, Wei-Ren Shen, Takahiro Noguchi, Yasuhiko Nara, Itaru Mizoguchi

**Affiliations:** Division of Orthodontics and Dentofacial Orthopedics, Department of Translational Medicine, Tohoku University Graduate School of Dentistry, 4-1 Seiryo-machi, Aoba-ku, Sendai 980-8575, Japan

## Abstract

Osteocytes are abundant cells in bone, which contribute to bone maintenance. Osteocytes express receptor activator of nuclear factor kappa-B ligand (RANKL) and regulate osteoclast formation. Orthodontic tooth movement (OTM) occurs by osteoclast resorption of alveolar bone. Osteocyte-derived RANKL is critical in bone resorption during OTM. Additionally, tumor necrosis factor-*α* (TNF-*α*) is important in osteoclastogenesis during OTM. Sclerostin has been reported to enhance RANKL expression in the MLO-Y4 osteocyte-like cell line. This study investigated the effect of TNF-*α* on sclerostin expression in osteocytes during OTM. *In vitro* analysis of primary osteocytes, which were isolated from DMP1-Topaz mice by sorting the Topaz variant of GFP-positive cells, revealed that *SOST* mRNA expression was increased when osteocytes were cultured with TNF-*α* and that *RANKL* mRNA expression was increased when osteocytes were cultured with sclerostin. Moreover, the number of TRAP-positive cells was increased in osteocytes and osteoclast precursors cocultured with sclerostin. *In vivo* analysis of mouse calvariae that had been subcutaneously injected with phosphate-buffered saline (PBS) or TNF-*α* revealed that the number of TRAP-positive cells and the percentage of sclerostin-positive osteocytes were higher in the TNF-*α* group than in the PBS group. Furthermore, the level of *SOST* mRNA was increased by TNF-*α*. As an OTM model, a Ni-Ti closed-coil spring connecting the upper incisors and upper-left first molar was placed to move the first molar to the mesial direction in wild-type (WT) mice and TNF receptor 1- and 2-deficient (TNFRsKO) mice. After 6 days of OTM, the percentage of sclerostin-positive osteocytes on the compression side of the first molar in TNFRsKO mice was lower than that in WT mice. In this study, TNF-*α* increased sclerostin expression in osteocytes, and sclerostin enhanced RANKL expression in osteocytes. Thus, TNF-*α* may play an important role in sclerostin expression in osteocytes and enhance osteoclast formation during OTM.

## 1. Introduction

Osteocytes, derived from osteoblasts, comprise 90%–95% of cells within bone tissue; they are stellate shaped and construct an intercellular network [1]. Multiple studies have shown that osteocytes play a central role as mechanosensory cells within bone [2, 3]; notably, osteocytes regulate bone remodeling by sensing mechanical stimulation and expression of various genes and proteins [4, 5].

Osteoclasts are large multinucleated cells derived from hematopoietic stem cells, which are responsible for bone resorption. Receptor activator of nuclear factor kappa-B ligand (RANKL) and macrophage colony stimulating factor (M-CSF) are known as essential factors for osteoclastogenesis [6]. Tumor necrosis factor-*α* (TNF-*α*) also affects osteoclastogenesis and can induce differentiation of osteoclast precursors into osteoclasts both *in vitro* [7–9] and *in vivo* [10, 11]. TNF-*α* is reportedly detected in response to excessive orthodontic force in rat periodontal tissues [12]. Moreover, TNF-*α* has been shown to play an important role in osteoclastogenesis during orthodontic tooth movement (OTM) [13–15].

Recently, two research groups found that osteocytes express RANKL and have a major role in osteoclastogenesis [16, 17]. Therefore, they have been proposed as a new therapeutic target for the treatment of osteoporosis [18–20]. In OTM, osteoclastic bone resorption occurs on the compression side, whereas osteoblastic bone formation occurs on the tension side [21]. In a study of OTM in transgenic mice, osteocytes were ablated by injecting diphtheria toxin into transgenic mice expressing diphtheria toxin receptors on the surface of osteocytes; subsequently, both the tooth movement distance and the number of osteoclasts in the compression side significantly decreased [22]. Recent investigations have demonstrated that osteocyte RANKL expression plays a key role in alveolar bone remodeling during OTM, using mice that specifically lack RANKL in osteocytes [23].

Sclerostin, which is encoded by the *SOST* gene, is a secreted glycoprotein that is primarily expressed in osteocytes; notably, it is an important negative regulator of bone homeostasis through inhibition of bone formation by osteoblasts [24]. Sclerostin binds to LRP5/6 as an antagonist of canonical Wnt signaling, thereby leading to inhibition of bone formation [25]. Sclerostin has been reported to increase RANKL expression in the MLO-Y4 osteocyte-like cell line, thereby promoting osteoclast formation [26]. TNF-*α* has been shown to upregulate sclerostin expression in MLO-Y4 cells [27]; consistent with this finding, the use of a TNF-*α* antagonist was able to diminish RANKL and sclerostin expression in the osteocytes of diabetic rats with periodontitis [28]. In general, mechanical stimulation to the bone is thought to reduce sclerostin expression in osteocytes [29]. However, some studies have shown that sclerostin expression can be increased by mechanical stimulation in mouse models of OTM [30–32]. The interaction between mechanical stimulation and sclerostin expression in osteocytes remains unclear. In addition, there has been no report regarding the effect of TNF-*α* on the expression of sclerostin in primary osteocytes.

In the present study, we investigated the influence of TNF-*α* on the expression of sclerostin in primary osteocytes, then examined how TNF-*α* affects the expression of sclerostin in osteocytes during OTM.

## 2. Materials and Methods

### 2.1. Mice and Reagents

C57BL/6J (wild-type: WT) mice were purchased from CLEA Japan Inc. (Tokyo, Japan). B6;129S-*Tnfrsf1a^tm1Imx^* (p55, TNFR1-deficient) *Tnfrsf1b^tm1Imx^*/J (p75, TNFR2-deficient) (TNFRsKO) mice and C57BL/6-Tg(Dmp1-Topaz)1lkal/J mice were purchased from The Jackson Laboratory (Bar Harbor, ME, USA). The protocols for all animal procedures were performed in accordance with Tohoku University regulations.

Recombinant mouse TNF-*α* for *in vivo* experiments was prepared in our laboratory as previously described [10]. Recombinant mouse TNF-*α* and recombinant human sclerostin (rhSCL) for *in vitro* experiments were purchased from R&D Systems (Minneapolis, MN, USA).

### 2.2. Preparation of Osteocytes

We followed a previously described method for osteocyte isolation, with minor modifications [33]. Calvariae of 5–6-day-old Dmp1-Topaz mice were dissected. A 0.2% (*w*/*v*) collagenase (Wako, Japan) solution was prepared immediately before use in isolation buffer (70 mM NaCl, 10 mM NaHCO_3_, 60 mM sorbitol, 3 mM K_2_HPO_4_, 1 mM CaCl_2_, 0.1% (*w*/*v*) bovine serum albumin (BSA), 0.5% (*w*/*v*) glucose, and 25 mM 4-(2-hydroxyethyl)-1-piperazineethanesulfonic acid (HEPES)). A 5 mM ethylenediaminetetraacetic acid (EDTA; Wako) solution was prepared with 0.1% BSA in phosphate-buffered saline (PBS) and sterilized by filtration through a 0.2 *μ*m filter. Calvariae were incubated in collagenase solution for 20 min or in EDTA solution for 15 min at 37°C with agitation, and the digests were collected. Incubations were performed as follows: collagenase (fraction 1), EDTA (fraction 2), collagenase (fraction 3), collagenase (fraction 4), and EDTA (fraction 5). Cell fractions 2–5 were cultured overnight, and adherent cells were collected with a trypsin-EDTA solution (Sigma-Aldrich, Japan). The cell suspension was then filtered through a 40 *μ*m nylon cell strainer (Falcon, USA). Topaz-positive osteocytes were isolated from cell fractions 2–5 by using a cell sorter (FACSAria II). Cell fraction 2 was used, as it comprised the osteoblast-rich fraction. Cells were cultured in alpha minimum essential medium (*α*-MEM) (Wako, Japan) containing 10% fetal bovine serum (FBS) and 1% penicillin-streptomycin (PS; 100 IU/mL penicillin G and 100 *μ*g/mL streptomycin).

### 2.3. Preparation of Osteoclast Precursors

C57BL/6J mice were sacrificed; then, femora and tibiae were immediately dissected. The epiphyses of these bones were cut, and the bone marrow was then flushed out with *α*-MEM (Wako, Japan). The cell suspension was filtered through a 40 *μ*m nylon cell strainer (Falcon). The obtained cells were cultured in *α*-MEM containing 10% FBS, 1% PS, and M-CSF for 3 days. Adherent cells were collected with a trypsin-EDTA solution (Sigma-Aldrich) and used as osteoclast precursors [34].

### 2.4. Preparation of RNA and Real-Time Reverse Transcription Polymerase Chain Reaction (RT-PCR) Analysis

For *in vitro* analysis, osteocytes were incubated in 24-well plates in culture medium that was supplemented with TNF-*α* (0 ng/mL and 100 ng/mL) for 1 day or 3 days, and with rhSCL (0 ng/mL, 1 ng/mL, 10 ng/mL, and 100 ng/mL) for 2 days. Total RNA from osteocytes was isolated with an RNeasy Mini Kit (Qiagen, USA).

For *in vivo* analysis, PBS or TNF-*α* (3.0 *μ*g/day) was injected into the supracalvarial region of C57BL/6J mice, once daily for 5 days. After 5 days, the mice were sacrificed and calvariae were immediately removed and frozen in liquid nitrogen. Subsequently, calvariae were homogenized using a Micro Smash MS-100R (TOMY SEIKO Co. Ltd., Japan) and then centrifuged in TRIzol Reagent (Invitrogen, USA). Total RNA was extracted from samples with an RNeasy Mini Kit (Qiagen), in accordance with the manufacturer's protocol.

cDNA was synthesized by using SuperScript IV Reverse Transcriptase (Invitrogen). Gene expression levels were analyzed by real-time RT-PCR in a Thermal Cycler Dice Real Time System (Takara, Japan) using TB Green Premix Ex Taq II (Takara, Japan). The PCR cycling conditions were as follows: initial denaturation stage (95°C for 30 sec), amplification stage (50 amplification cycles with each cycle composed of a denaturation step of 95°C for 5 s and an annealing step of 60°C for 30 s), and final dissociation stage (a cycle composed of 95°C for 15 s, 60°C for 30 s, and 95°C for 15 s). Glyceraldehyde 3-phosphate dehydrogenase (*GAPDH*) mRNA was used to normalize each gene expression levels. The primers were as follows: *GAPDH*, 5′-GGTGGAGCCAAAAGGGTCA-3′ and 5′-GGGGGCTAAGCAGTTGGT-3′; *DMP1*, 5′-ACCACACGGACAGCAGTGAATC-3′ and 5′-CCTCATCGCCAAAGGTATCATCTC-3′; *SOST*, 5′-AGCCTTCAGGAATGATGCCAC-3′ and 5′-CTTTGGCGTCATAGGGATGGT-3′; *RANKL*, 5′-CCTGAGGCCAGCCATTT-3′ and 5′-CTTGGCCCAGCCTCGAT-3′; and *OPG*, 5′-ATCAGAGCCTCATCACCTT-3′ and 5′-CTTAGGTCCAACTACAGAGGAAC-3′.

### 2.5. Coculture of Osteocytes and Osteoclast Precursors for Osteoclast Formation

Osteocytes (2 × 10^4^ cells) and osteoclast precursors (5 × 10^4^ cells) were cocultured in 200 *μ*L *α*-MEM containing 10% FBS and 1% PS in a 96-well plate, in the presence of 10^−8^ M 1,25-dihydroxyvitamin D3 (Sigma-Aldrich) and 10^−6^ M prostaglandin E2 (Sigma-Aldrich) with and without rhSCL (100 ng/mL). Medium was changed on the second day.

After 5 days, cell cultures were fixed in a 4% paraformaldehyde solution for 30 min, then permeabilized with 0.2% Triton X-100 for 1 h at room temperature. Tartrate-resistant acid phosphatase (TRAP) staining solution was prepared to visualize osteoclasts by mixing acetate buffer (pH 5.0), naphthol AS-MX phosphate (Sigma-Aldrich), Fast Red Violet LB Salt (Sigma-Aldrich), and 50 mM sodium tartrate. TRAP-positive cells with three or more nuclei were regarded as osteoclasts and were counted under a light microscope [34].

### 2.6. Experimental Tooth Movement

OTM was performed as described previously [35]. In brief, 8–12-week-old male WT mice and TNFRsKO mice were anesthetized and a nickel titanium (Ni-Ti) closed-coil spring (TOMY SEIKO Co. Ltd.) was attached between the upper incisor and left first molar. The appliance was fixed with a stainless-steel wire (0.01 mm diameter) to the hole drilled in the upper anterior alveolar bone, then tied to the first molar. The first molar was moved in the mesial direction with a force of 10 g.

### 2.7. Preparation for Histological Observation

Harvested calvariae and maxillae were fixed overnight in 4% paraformaldehyde at 4°C. Samples were decalcified in 14% EDTA for 3 days (calvariae) or 1 month (maxillae) at 4°C. After dehydration, samples were embedded in paraffin and cut in coronal sections of 5 *μ*m (calvariae) and horizontal sections of 4 *μ*m (maxillae) thickness. Maxillae sections were taken at approximately 150 *μ*m from the root branch of the upper-left first molar.

To confirm osteoclast formation, calvariae paraffin sections were stained with TRAP solution, then counterstained with hematoxylin. TRAP-positive cells with three or more nuclei were regarded as osteoclasts. The numbers of TRAP-positive cells were counted and averaged within the suture of sagittal sutures [34].

For immunohistochemistry, paraffin sections were deparaffinized, rehydrated, and then treated with 3% H_2_O_2_ for 15 min. Thereafter, sections were blocked with 5% skim milk for 30 min at 37°C and treated with anti-SOST polyclonal goat antibody AF1589 (1 : 50 diluted in blocking buffer; R&D Systems) overnight at 4°C. After sections had been rinsed, they were processed with VECTASTAIN Elite ABC Kit PK-6105 (Vector Laboratories Inc., USA) and treated with 3,3′-diaminobenzidine (DAB). Hematoxylin was used for counterstaining [36]. We confirmed that the percentage of sclerostin-positive osteocytes was within the range of 400 *μ*m × 200 *μ*m from the mesial periodontal ligament on the compression side of the distobuccal root of the upper-left first molar.

### 2.8. Statistical Analysis

All values are presented as mean ± standard deviation. Statistical analyses were performed using Student's *t*-test. *P* < 0.05 was considered to be statistically significant.

## 3. Results

### 3.1. Isolation and Characterization of Osteocytes

**Figure 1:**
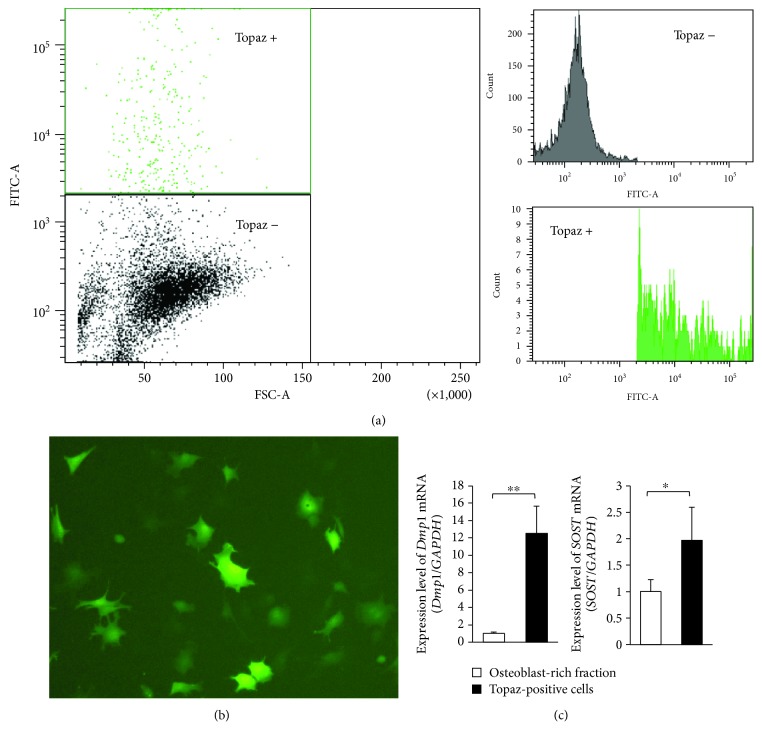
Characteristics of isolated Topaz-positive cells. (a) Sorting of Topaz-positive cells with the FACSAria II Cell Sorter. (b) Morphology of Topaz-positive cells. (c) Expression levels of *DMP1* and *SOST* mRNA in the osteoblast-rich fraction (fraction 2) and in all Topaz-positive cells (*n* = 4; ^∗^ *P* < 0.05 and ^∗∗^ *P* < 0.01).

To isolate high-purity osteocytes, we sorted Topaz-positive and negative cells from cell fractions 2–5 from calvariae of Dmp1-Topaz mice (Figure 1(a)). Topaz-positive cells exhibited a stellate-shaped morphology (Figure 1(b)). We confirmed that expression of osteocyte-specific genes, such as *Dmp1* and *SOST*, was higher in Topaz-positive cells than in osteoblasts (Figure 1(c)).

### 3.2. TNF-*α* Enhances Sclerostin Expression of Osteocytes and Sclerostin Promotes Osteoclastogenesis In Vitro

**Figure 2:**
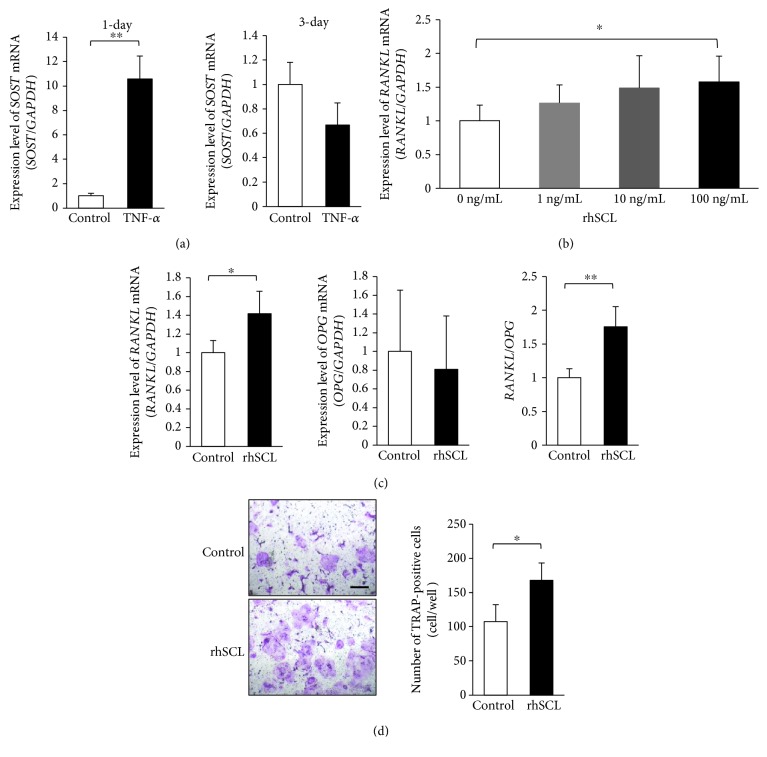
Tumor necrosis factor alpha (TNF-*α*) enhanced sclerostin expression in primary osteocytes, and sclerostin enhanced osteoclastogenesis *in vitro*. (a) Expression of *SOST* mRNA in osteocytes, analyzed by real-time reverse transcription polymerase chain reaction (RT-PCR). Total RNA in osteocytes was isolated from osteocytes cultured with or without TNF-*α* for 1 or 3 days (*n* = 4; ^∗∗^ *P* < 0.01). (b) Expression of *RANKL* mRNA in osteocytes, analyzed by real-time RT-PCR. Total RNA from osteocytes was isolated from osteocytes cultured with recombinant human sclerostin (rhSCL) (0 ng/mL, 1 ng/mL, 10 ng/mL, and 100 ng/mL) for 2 days (*n* = 4; ^∗^ *P* < 0.05). (c) Expression levels of *RANKL* and *OPG* mRNA, as well as *RANKL*/*OPG* ratio in osteocytes, analyzed by real-time RT-PCR. Total RNA was isolated from osteocytes cultured with or without rhSCL (100 ng/mL) for 2 days (*n* = 4; ^∗^ *P* < 0.05 and ^∗∗^ *P* < 0.01). (d) Microscopic photos and the numbers of tartrate-resistant acid phosphatase- (TRAP-) positive cells within a coculture of osteocytes and osteoclast precursors, with or without rhSCL, in the presence of vitamin D3 and prostaglandin E2. Scale bars = 100 *μ*m (*n* = 4; ^∗^ *P* < 0.05).

We performed real-time RT-PCR to analyze *SOST* mRNA expression of osteocytes that were treated with TNF-*α in vitro*. Compared with control (untreated) osteocytes, *SOST* mRNA expression increased in 1-day culture of TNF-*α*-treated osteocytes; however, there was no significant difference in 3-day culture (Figure 2(a)). Furthermore, to investigate the relationship between sclerostin and osteoclastogenesis, we cultured osteocytes with rhSCL (0 ng/mL, 1 ng/mL, 10 ng/mL, and 100 ng/mL) for 2 days. Compared with osteocytes treated with 0 ng/mL rhSCL, *RANKL* mRNA expression was significantly increased in osteocytes treated with 100 ng/mL rhSCL; in contrast, *OPG* mRNA expression showed no significant difference on the basis of rhSCL treatment (Figures 2(b) and 2(c)). *RANKL*/*OPG* ratio was significantly increased in osteocytes treated with 100 ng/mL rhSCL (Figure 2(c)). The effect on osteoclast formation was analyzed by coculture of osteocytes and osteoclast precursors. The number of TRAP-positive cells increased among osteocytes that were treated with rhSCL (Figure 2(d)).

### 3.3. TNF-*α* Induced Osteoclastogenesis and Sclerostin Expression of Osteocytes In Vivo

**Figure 3:**
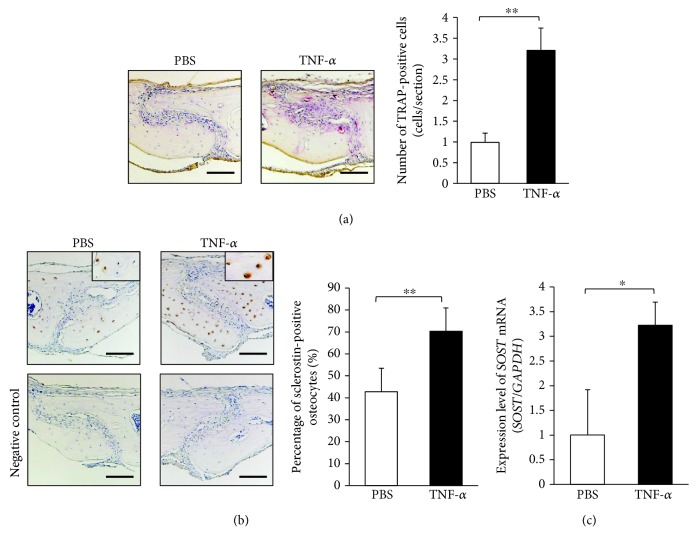
TNF-*α* enhanced osteoclastogenesis and sclerostin expression in osteocytes *in vivo*. (a) Images: histological sections of calvariae were obtained from wild-type mice after 5 days of daily supracalvarial administration of phosphate-buffered saline (PBS) or 3.0 *μ*g/day tumor necrosis factor alpha (TNF-*α*). Sections were stained with tartrate-resistant acid phosphatase (TRAP); cells stained red are regarded as TRAP-positive. Scale bars = 100 *μ*m. Graph: the numbers of TRAP-positive cells on the calvariae (*n* = 4; ^∗∗^ *P* < 0.01). (b) Images: sections were immunolocalized with antibodies specific for sclerostin, then counterstained with hematoxylin. Scale bars = 100 *μ*m. Graph: the numbers of sclerostin-positive osteocytes on the calvariae (*n* = 4; ^∗∗^ *P* < 0.01). (c) *SOST* mRNA levels in mouse calvariae detected using real-time reverse transcription polymerase chain reaction.

To investigate the effect of TNF-*α in vivo*, we subcutaneously injected PBS or TNF-*α* into mice cranial part for 5 days. The number of TRAP-positive cells in the suture of histological sections from mice in the TNF-*α*-injected group was significantly increased, compared with that of mice in the PBS-injected group (Figure 3(a)). Immunohistochemical analysis showed that the percentage of sclerostin-positive osteocytes was higher in sections from mice in the TNF-*α* group than in sections from mice in the PBS group (Figure 3(b)). Real-time RT-PCR results also revealed that *SOST* mRNA expression levels were higher among mice in the TNF-*α* group than among mice in the PBS group (Figure 3(c)).

### 3.4. TNF-*α* Affects Sclerostin Expression in Osteocytes during OTM

**Figure 4:**
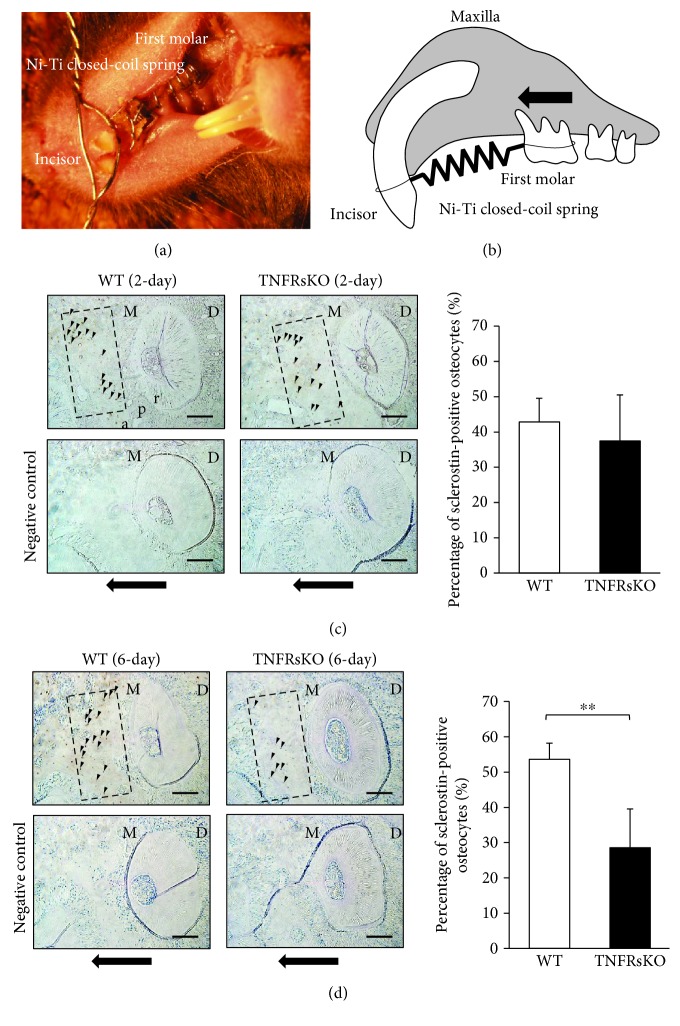
Tumor necrosis factor alpha (TNF-*α*) affects sclerostin expression in osteocytes during orthodontic tooth movement (OTM). (a) Intraoral photograph of the OTM appliance. (b) Schematic diagram of orthodontic tooth movement of the upper-left first molar to the mesial side. The black arrow indicates the direction of orthodontic force. (c, d) Images: the percentage of sclerostin-positive osteocytes was examined in the range of 400 *μ*m × 200 *μ*m from the mesial periodontal ligament on the compression side around 150 *μ*m from the distobuccal root branch of the upper-left first molar after 2-day (c) or 6-day OTM (d). Arrowheads indicate the sclerostin-positive cells. Black arrows indicate the direction of orthodontic force. M, mesial side; D, distal side; a, alveolar bone; p, periodontal ligament; r, root. Scale bars = 100 *μ*m. Graphs: corresponding percentages of sclerostin-positive osteocytes during OTM (*n* = 4; ^∗∗^ *P* < 0.01).

As an OTM model, a Ni-Ti closed-coil spring was fixed between the upper anterior alveolar bone and the upper-left first molar to move the first molar in the mesial direction in both WT and TNFRsKO mice (Figures 4(a) and 4(b)). Sections underwent immunohistochemical staining, and sclerostin-positive osteocytes were evaluated under an optical microscope. This analysis revealed that the percentage of sclerostin-positive osteocytes in TNFRsKO mice was less than that of WT mice after 6 days of OTM, whereas there was no significant difference between groups after 2 days of OTM (Figures 4(c) and 4(d)).

## 4. Discussion

In this study, we found that stimulation with TNF-*α* increased sclerostin expression in primary osteocytes. In addition, sclerostin enhanced RANKL-induced osteoclast formation *in vitro*. Furthermore, stimulation with TNF-*α* enhanced the expression of sclerostin in osteocytes *in vivo*. Notably, we evaluated the effect of TNF-*α* on the expression of sclerostin in osteocytes during OTM; we showed that sclerostin was increased in WT mice but not in TNFRsKO mice during OTM. Our findings suggest that TNF-*α* plays an important role in increasing the expression of sclerostin in osteocytes and enhancing osteoclast formation during OTM. This experiment is the first to demonstrate an interaction between TNF-*α* stimulation and sclerostin expression in osteocytes during OTM.

Osteocytes are the most abundant cells in bone tissue, but their role in bone remodeling has been unclear because they are embedded in a mineralized matrix and are difficult to isolate. Notably, the murine long bone osteocyte Y4 (MLO-Y4) cell line was generated to study osteocyte function [37]. While MLO-Y4 cells are osteocyte-like, they do not necessarily reproduce similar reactions to osteocytes *in vivo*. Although there is an established method for isolation of primary osteocytes—the so-called “conventional method”—it produces approximately 60% osteocytes [38]. Recently, a new method for isolating primary osteocytes was established using mice with osteocyte-specific expression of GFP [16]; this method enabled isolation of osteocytes with greater purity than that demonstrated by the conventional method. Therefore, we used this new method to isolate primary osteocytes from DMP1-Topaz mice, which express the Topaz variant of GFP under the direction of the mouse *Dmp1* promoter. In the conventional method, fraction 5 is considered to be the osteocyte-rich fraction [38]. We evaluated the ratio of Topaz-positive cells in fraction 5, and found that it was <10%. In addition, we evaluated the characteristics of Topaz-positive cells by assessment of the osteocyte markers *Dmp1* and *SOST*, and found high expression of osteocyte markers. These results suggested that the use of Dmp1-Topaz mice enables purification of more osteocytes than that enabled by the conventional method. Therefore, the Topaz-positive cells purified as osteocytes may closely represent the properties of osteocytes.

A few studies have reported the effect of TNF-*α* on sclerostin expression in osteocytes; for example, an increased level of TNF-*α* is associated with estrogen deficiency, which may stimulate sclerostin expression in osteocytes [39]. TNF-*α* has also been shown to upregulate sclerostin expression in MLO-Y4 cells [27]. In addition, treatment with a TNF-*α* antagonist could reduce sclerostin expression in osteocytes in diabetic rats with periodontitis [28]. In the present study, we cultured Topaz-positive cells as primary osteocytes, with or without TNF-*α*. Notably, 1-day culture with TNF-*α* increased sclerostin expression in primary osteocytes, compared with control osteocytes; however, 3-day culture with TNF-*α* did not increase sclerostin expression. Thus, we concluded that stimulation with TNF-*α* increased sclerostin expression in primary osteocytes in the initial stage of culture. Next, to analyze the effect of sclerostin on RANKL-induced osteoclastogenesis *in vitro*, we cultured primary osteocytes with various concentrations of sclerostin (0 ng/mL, 1 ng/mL, 10 ng/mL, and 100 ng/mL), and found a significant increase in the expression of *RANKL* mRNA with 100 ng/mL sclerostin. In the present study, *RANKL* mRNA expression was slightly increased with 10 ng/mL sclerostin, but there was no significant difference between 0 ng/mL and 10 ng/mL sclerostin. However, at 100 ng/mL sclerostin *RANKL* mRNA was significantly increased. This is in contrast to what was reported by Wijenayaka et al., where *RANKL* mRNA expression was significantly increased with 10 ng/mL sclerostin when added to culture of osteocyte-like cells compared to untreated control [26]. We can attribute the difference in response strength to the method used in obtaining osteocytes; while this study relied on primary osteocytes obtained from DMP1-Topaz mice through cell sorting, others used osteocytes differentiated from normal human bone-derived cells (NHBC) [26]. Other factors that can contribute to the variation in the strength of response are different culture conditions and different sources from which osteocytes were obtained. Moreover, the ratio of *RANKL*/*OPG* significantly increased upon stimulation with 100 ng/mL sclerostin. Sclerostin has been reported to stimulate RANKL expression in MLO-Y4 cells [26, 31]. Our experiments have revealed similar results in analyses of both primary osteocytes and MLO-Y4 cells. Moreover, when primary osteocytes and osteoclast precursors were cocultured, the number of TRAP-positive cells increased in the presence of sclerostin. Finally, sclerostin reportedly promotes osteoclast formation via RANKL expression in MLO-Y4 cells [26, 31]. Our results regarding primary cells in this study support the previous *in vitro* findings.

We previously reported that osteoclasts can be induced in calvariae in the presence of TNF-*α in vivo* [10, 11]. In the present study, we analyzed osteoclast formation and sclerostin expression in osteocytes by subcutaneous injection of TNF-*α* into mice cranial part for 5 days. First, we confirmed that stimulation with TNF-*α* induced osteoclast formation in calvariae by using TRAP staining, as in our previous studies [10, 11]. Next, we investigated the effect of TNF-*α* on sclerostin expression in osteocytes by using immunohistochemical analysis. We found that the percentage of sclerostin-positive osteocytes and the expression level of *SOST* mRNA both increased in the TNF-*α* group. Thus, TNF-*α* may enhance sclerostin expression in osteocytes *in vivo*.

The levels of TNF-*α* have been reported to increase in the gingival sulcus in humans during OTM [40, 41]. We previously showed that OTM was mediated by TNF-*α*, in studies of TNFRsKO mice [13, 15]. Here, we used TNFRsKO mice to assess the role of TNF-*α* in the induction of sclerostin in osteocytes during OTM. Orthodontic force was applied to the upper-left first molar in the mesial direction for 2 or 6 days. The percentage of sclerostin-positive osteocytes in the compression side was significantly reduced in TNFRsKO mice in the 6-day OTM group. Several studies have shown that high sclerostin expression is maintained for 5–7 days after the initiation of tooth movement [31, 32]. Importantly, our results indicated that TNF-*α* influenced sclerostin expression in osteocytes on day 6 of OTM. Moreover, recent investigation has shown that RANKL expression and osteoclast formation were decreased in SOST-deficient mice compared to wild-type mice at the compression side during OTM [31]. These results suggest that sclerostin affects osteoclastogenesis at the compression side during OTM.

## 5. Conclusions

**Figure 5:**
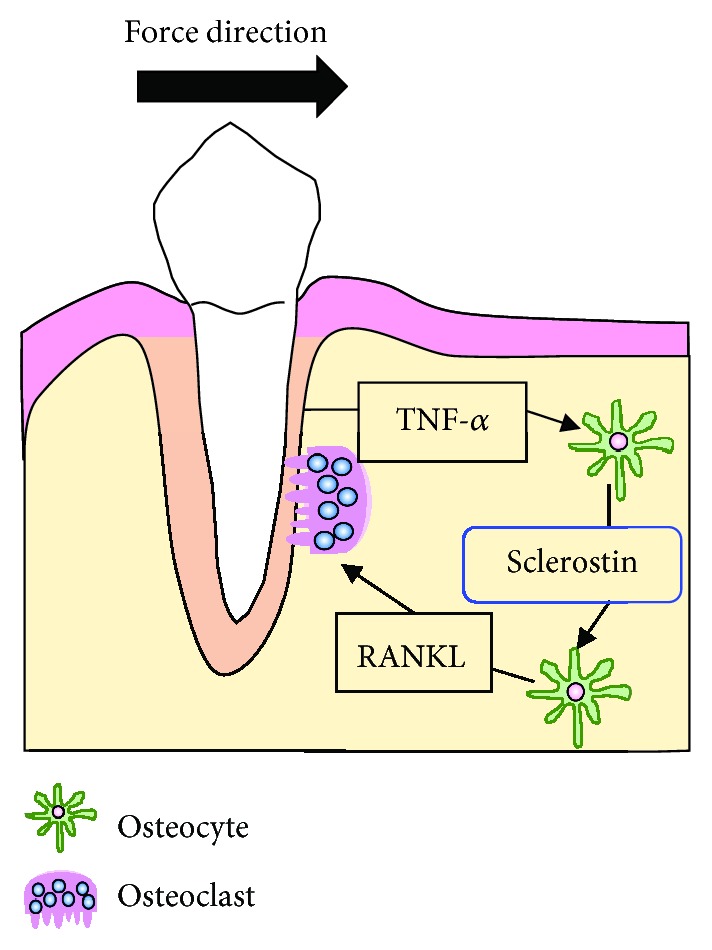
Schematic diagram explaining the role of tumor necrosis factor alpha (TNF-*α*) in inducing sclerostin expression in osteocytes on the compression side during orthodontic tooth movement (OTM). TNF-*α* enhances sclerostin expression in osteocytes; then, sclerostin increases the expression of receptor activator of nuclear factor kappa-B ligand (RANKL) by osteocytes. Hence, we concluded that osteoclast formation was enhanced by TNF-*α* through increased sclerostin expression in osteocytes on the compression side during OTM.

We found that TNF-*α* enhanced the expression of sclerostin in osteocytes, and that sclerostin-induced RANKL expression in osteocytes enhanced osteoclastogenesis during OTM. In conclusion, we have obtained evidence that TNF-*α* plays an important role on sclerostin expression in osteocytes on the compression side during OTM (Figure 5).

## Data Availability

The data used to support the findings of this study are included within the article.

## Supplementary Materials

Supplementary Materials The supplementary materials contain a figure with the analysis of TRAP-positive cells. To investigate the expression of calcitonin receptor (CTR) on TRAP-positive cells, TRAP staining and immunohistochemical staining were performed on the calvariae of C57BL/6J mice. CTR-positive cells were identified in the same area as multinucleated TRAP-positive cells in the serial section (Figure S1A). The number of CTR-positive cells in the suture of histological sections from mice in the TNF-*α*-injected group was significantly increased compared to that of mice in the PBS-injected group (Figure S1B). Similar results to TRAP staining (Figure 3(a)) were obtained with immunohistochemical staining of calcitonin receptor (Figure S1B).
