# Supplementary material for: Effect of TNF-α-Induced Sclerostin on Osteocytes during Orthodontic Tooth Movement
Source: J Immunol Res. 2019 Jun 24;2019:9716758. doi: 10.1155/2019/9716758 (PMC6612957; doi:10.1155/2019/9716758)
Supplement: Supplementary Materials — The supplementary materials contain a figure with the analysis of TRAP-positive cells. To investigate the expression of calcitonin receptor (CTR) on TRAP-positive cells, TRAP staining and immunohistochemical staining were performed on the calvariae of C57BL/6J mice. CTR-positive cells were identified in the same area as multinucleated TRAP-positive cells in the serial section (Figure S1A). The number of CTR-positive cells in the suture of histological sections from mice in the TNF-α-injected group was significantly increased compared to that of mice in the PBS-injected group (Figure S1B). Similar results to TRAP staining (Figure 3(a)) were obtained with immunohistochemical staining of calcitonin receptor (Figure S1B). [file 9716758.f1.pdf]

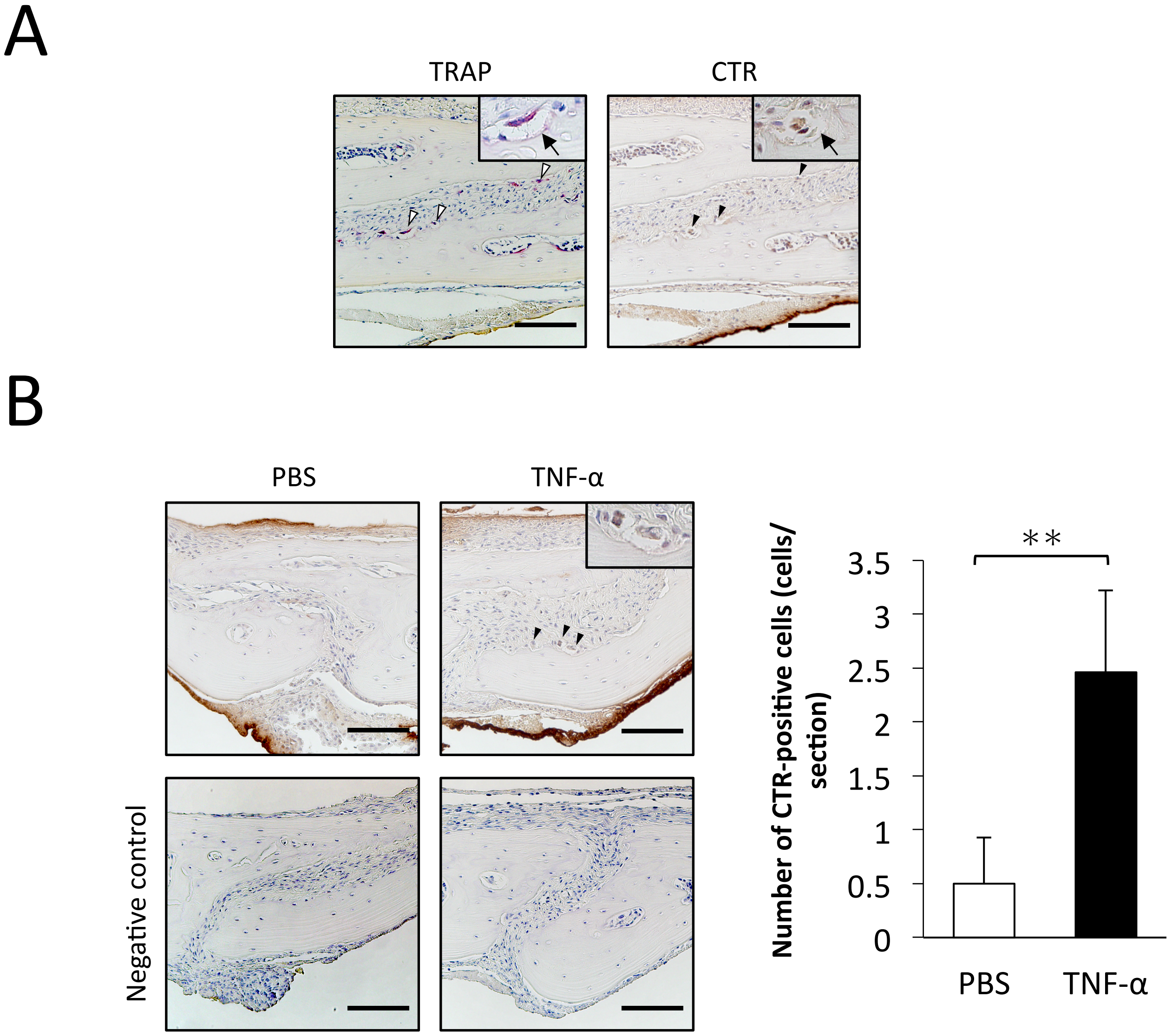

Figure S1: PBS or TNF-α (3.0 μg/day) was injected in the supracalvarial region of C57BL/6J mice, once daily for 5 days. Harvested calvariae were fixed overnight in 4% paraformaldehyde at 4°C. Samples were decalcified in 14% EDTA for 3 days at 4°C. After dehydration, samples were embedded in paraffin and cut in coronal sections of 5 μm thickness. For immunohistochemical staining, paraffin sections were deparaffinized, rehydrated, and then treated with 3% H_2O_2 for 15 min. Thereafter, sections were blocked with 5% skim milk for 30 min at 37°C and treated with anti-calcitonin receptor (CTR) antibody ab11042 (1:50 diluted in blocking buffer; R&D Systems) overnight at 4°C. After sections had been rinsed, they were processed with VECTASTAIN Elite ABC Kit PK-6101 (Vector Laboratories, USA) and treated with 3,3′-diaminobenzidine (DAB). Hematoxylin was used for counterstaining.

(A) Serial sections in which TNF-α (3.0 μg/day) was injected into calvariae of mice, once daily for 5 days. Left image is TRAP staining and right image is immunohistochemical staining. *Black arrow* indicates the bone resorption area. *White arrowheads* indicate multinucleated TRAP-positive cells. *Black arrowheads* indicate calcitonin receptor (CTR)-positive cells. Scale bars = 100 μm. (B) (Images) Histological sections of calvariae were immunolocalized with antibodies specific for calcitonin receptor. *Black arrowheads* indicate CTR-positive cells. Scale bars = 100 μm. (Graph) The numbers of CTR-positive cells on the calvariae (n = 4; $**P < 0.01$).
